# Fine-Grained Grape Leaf Diseases Recognition Method Based on Improved Lightweight Attention Network

**DOI:** 10.3389/fpls.2021.738042

**Published:** 2021-10-22

**Authors:** Peng Wang, Tong Niu, Yanru Mao, Bin Liu, Shuqin Yang, Dongjian He, Qiang Gao

**Affiliations:** ^1^College of Mechanical and Electronic Engineering, Northwest Agriculture and Forestry (A&F) University, Yangling, China; ^2^Key Laboratory of Agricultural Internet of Things, Ministry of Agriculture and Rural Affairs, Xianyang, China; ^3^Shaanxi Key Laboratory of Agricultural Information Perception and Intelligent Services, Xianyang, China; ^4^College of Information Engineering, Northwest Agriculture and Forestry (A&F) University, Yangling, China

**Keywords:** grape leaf diseases, diseases recognition, fine-grained image, attention mechanism, lightweight

## Abstract

Real-time dynamic monitoring of orchard grape leaf diseases can greatly improve the efficiency of disease control and is of great significance to the healthy and stable development of the grape industry. Traditional manual disease-monitoring methods are inefficient, labor-intensive, and ineffective. Therefore, an efficient method is urgently needed for real-time dynamic monitoring of orchard grape diseases. The classical deep learning network can achieve high accuracy in recognizing grape leaf diseases; however, the large amount of model parameters requires huge computing resources, and it is difficult to deploy to actual application scenarios. To solve the above problems, a cross-channel interactive attention mechanism-based lightweight model (ECA-SNet) is proposed. First, based on 6,867 collected images of five common leaf diseases of measles, black rot, downy mildew, leaf blight, powdery mildew, and healthy leaves, image augmentation techniques are used to construct the training, validation, and test set. Then, with ShuffleNet-v2 as the backbone, an efficient channel attention strategy is introduced to strengthen the ability of the model for extracting fine-grained lesion features. Ultimately, the efficient lightweight model ECA-SNet is obtained by further simplifying the network layer structure. The model parameters amount of ECA-SNet 0.5× is only 24.6% of ShuffleNet-v2 1.0×, but the recognition accuracy is increased by 3.66 percentage points to 98.86%, and FLOPs are only 37.4 M, which means the performance is significantly better than other commonly used lightweight methods. Although the similarity of fine-grained features of different diseases image is relatively high, the average F1-score of the proposed lightweight model can still reach 0.988, which means the model has strong stability and anti-interference ability. The results show that the lightweight attention mechanism model proposed in this paper can efficiently use image fine-grained information to diagnose orchard grape leaf diseases at a low computing cost.

## Introduction

Grape leaf disease is the main factor that causes a large-scale reduction in orchards and restricts the healthy and stable development of the grape industry. Realizing real-time dynamic monitoring of orchard diseases is of great significance for the early prevention and control of orchard diseases and the cultivation of disease-resistant varieties. In recent years, with the development of computer vision technology and the continuous improvement of computing power, researchers have used deep learning methods in the field of crop disease diagnosis and have achieved remarkable results in general disease recognition tasks (Lu et al., 2017; Priyadharshini et al., 2019; Chen et al., 2020; Liu et al., 2020). For instance, Ma J. et al. (2018) proposed a deep convolutional neural network to identify three types of cucumber diseases and achieved an accuracy of 93.4%. Liu et al. (2018) proposed a network based on AlexNet and GoogLeNet, which used deep learning to diagnose apple leaf diseases for the first time. The accuracy on the test set reached 97.62%, which was better than traditional machine learning methods. Ferentinos (2018) tested five classical convolutional neural networks to identify plant leaf diseases, and the results showed that all of them can achieve ideal accuracy. Although the convolution neural network-based classification models mentioned above can achieve superior recognition results, it has the imperfection of highly dependent on the hardware performance of the device. The huge amount of network parameters leads to huge computational overhead, which cannot be afforded by ordinary devices, and it is difficult to deploy to the terminals for promotion.

In view of the high computational cost of large-scale models, many scholars have carried out pieces of lightweight model research. Xception (Chollet, 2017) was a lightweight model improved by Google based on the Inception-v3 (Szegedy et al., 2016). The deep separable convolution was used to reduce the parameters, but the computational cost was increased. Compared with traditional convolutional neural networks, while using deep separable convolution, MobileNet-v1 (Howard et al., 2017) introduces two hyperparameters that control the number of convolution kernels and the resolution of the input image. This model leveraged a stack layer structure; although the number of parameters was reduced, there still exists the model degradation problem. Sandler et al. (2018) suppressed the degradation of the model by introducing the Inverted Residuals structure. By using a linear activation function to effectively retain low-dimensional features, the parameters of the model were further reduced, and the accuracy was improved. Howard et al. (2019) added the Squeeze-Excitation module (Hu et al., 2018) to the Inverted Residual structure to endow the ability of the model to focus on key feature channels. The lightweight model structure was designed to be flexible and efficient, which may greatly reduce the calculation cost and easy to be applied on mobile terminals, including smartphones, embedded devices, etc. With its own advantages, the application of lightweight networks in the field of crop disease identification has also made some progress. Chao et al. (2020) combined DenseNet and Xception strategies and proposed XDNet to identify five apple leaf diseases. The model recognition effect was preferable, and the amount of parameters was not high. Tang et al. (2020) introduced the SE module into the ShuffleNet network and proposed a lightweight convolutional neural network. The public data containing four types of grape diseases were used to evaluate the network performance; the accuracy of the training set can reach 99.14%. Bi et al. (2020) used the MobileNet network to identify two different apple diseases and compared with other models in terms of efficiency and accuracy to verify the effectiveness of the network. The above pieces of research have opened up a new way for the promotion of low-cost models; however, there are still problems that existing lightweight method cannot achieve pleasant and stable performance on a fine-grained image recognition task. Ramcharan et al. (2019) used a mobile device equipped with a lightweight model to diagnose cassava diseases in the field and found that the different angles, brightness, and changes in different diseases will affect the accuracy of the model.

Different from general image recognition tasks, the key information of the local area plays a decisive role in the classification decision in fine-grained image recognition. So, how to make full use of the effective information is the key to improve the performance on fine-grained recognition. Under the influence of many interference factors, the feature differences among different subclasses of a specific category of images may be small, or the feature differences among different objects in the same subclass may be large, which increases the difficulty of fine-grained image classification. Therefore, the fine-grained disease image recognition under the complex background has higher requirements for the comprehensive performance of the model. The fine-grained disease image identification method based on visual attention can effectively focus on the region of interest and improve the recognition performance of the model. In recent years, it has been widely used in image classification, object detection, and other fields and has achieved excellent results. Yang et al. (2020) proposed an attention mechanism that effectively used the key information of images and established the image classification model for 14 different crops based on transfer learning. The model was trained and tested with the PlantVillage public data set. The test results show that the F1-score of the proposed model can reach 0.93. Mi et al. (2020) introduced the attention mechanism into DenseNet to identify six different grades of wheat stripe rust and found that the performance of the model with attention mechanism can be significantly improved.

Inspired by the above research, a new lightweight model for fine-grained grape leaf disease recognition is proposed in this paper. The main innovations and contributions are summarized as below:

(1) A new grape leaf disease data set is established, and the fine-grained grape leaf diseases image datasets (FGGLDIs, namely FGDs) are generated *via* image enhancement techniques. The image enhancement techniques are used to simulate grape leaf disease images collected under complex environment conditions, enhance the generalization performance of the model, avoid over-fitting problems in the training process, and lay the foundation for the popularization of the model.
(2) In this paper, the cross-channel interaction strategy without dimensionality reduction is introduced into the lightweight network, and a new fine-grained recognition model of grape disease images based on the attention mechanism is proposed. First, deep separable convolution is used to reduce the overall amount of parameters of the model. Second, the efficient channel attention (ECA) is embedded into the ShuffleNet infrastructure and implemented through one-dimensional convolution. Finally, a method of adaptive selection of the size of the one-dimensional convolution kernel is adopted to determine the coverage of the cross-channel interaction. The method proposed in this paper can effectively reduce the complexity while maintaining the performance of the model, realize the effective fusion of multichannel features, and strengthen the ability of the model to learn important and fine-grained information in the lesion area.

The remainder of the paper is organized as follows: In *Materials and Methods*, the structure information of the data set is introduced, and FGDs is generated by using data enhancement techniques. The model structure and the test process mentioned in this article are discussed in detail. *Results and Discussion* presents the test method to evaluate the performance of the model and analyzes the test results. The model attention and fine-grained feature learning are also displayed through heat maps and fine-grained feature maps, respectively. The *Conclusion* summarizes the work of this article.

## Materials and Methods

This section introduces the materials and methods used in the study, including the collected grape leaf disease image data, FGDs established through image enhancement techniques, relevant lightweight network, and detailed structure of the proposed model.

### Data Acquisition

The original data set used in this study contains a total of 6,867 images of grape leaf disease from two parts. First, 3,388 images of powdery mildew, downy mildew, and healthy leaves are collected in the field of the grape planting experimental station of Northwest A&F University, Shaanxi Province, China. In different weather conditions and different time periods (sunny, cloudy, morning, noon, and evening), the MI 9 smartphone is used to shoot from different angles and directions. Then, a total of 3,479 black measles, black rot, and leaf blight are collected from the public data set. Through the above work, an original data set of common grape diseases is established.

**Figure 1:**
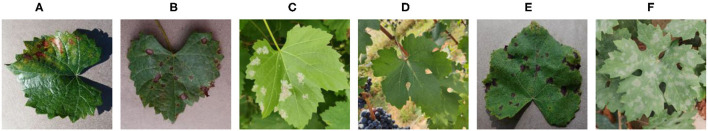
Examples of grape leaf images. **(A)** Black measles, **(B)** Black rot, **(C)** Downy mildew, **(D)** Healthy, **(E)** Leaf blight, and **(F)** Powdery mildew.

Figure 1 shows a random sample of each category of the data set. It can be seen from the examples that there are a large amount of complex background (Figures 1C,D,F) and pure-color background (Figures 1A,B,E) images in the data set, which has high requirements for the comprehensive performance of the model. In Figure 1, black measles (Figure 1A), black rot (Figure 1B), and leaf blight spots (Figure 1E) have a high degree of similarity. When the leaves are onset, brown spots are produced, which gradually expand into nearly circular spots with edges appearing dark brown. Downy mildew (Figure 1C) early disease spots are dense white frost-like objects, and the shape of the disease spots is usually irregular polygonal when restricted by leaf veins. When the disease is severe in the later stage, the leaves will fall off early. Powdery mildew (Figure 1F) leaves are covered with off-white powder, similar to downy mildew symptoms; both of which form clusters of lesions locally and are not easily distinguishable by the naked eye. Therefore, the above mentioned classification of different diseases can be expressed as a problem of fine-grained image classification.

### Image Dataset Augmentation

In the orchards, grape leaves grow in different positions with different shapes, and there are interference factors such as weather and shooting angles. So, the data set need to be expanded to avoid over-fitting during the training process. Before performing image data augmentation, 100 images were randomly selected from each category in the original data set, a total of 600 images, and then, adding Gaussian noise, rotated left 90°, rotated right 90°, vertically flipped, and weakened sharpness, respectively, forming an enhanced robustness test data set (RTD), containing 3,000 images. Gaussian blur, contrast enhancement by 30% and decrease by 30%, and brightness enhancement by 30% and decrease by 30% are adopted to the remaining images in the original data set to simulate weather interference. Then, rotating the image by 90°, 270°, horizontal flip, and vertical flip to simulate the disturbance of different shooting angles and the FGDs is established. Table 1 shows the detailed structure information of FGDs. After the model was trained, RTD was used to test the model to verify the effect of model training. The above work provides a data basis for model training.

**Table 1:** The structure of FGDs.

**Categories**	**Black measles**	**Black rot**	**Downy mildew**	**Healthy**	**Leaf blight**	**Powdery mildew**
Training set	8,664	8,640	8,080	8,112	8,128	8,512
Validation set	2,166	2,160	2,020	2,028	2,032	2,128
Total	10,830	10,800	10,100	10,140	10,160	10,640
Proportion	0.1728	0.1723	0.1612	0.1618	0.1621	0.1698

### ECA-SNet Network

#### Relevant Lightweight Network

**Figure 2:**
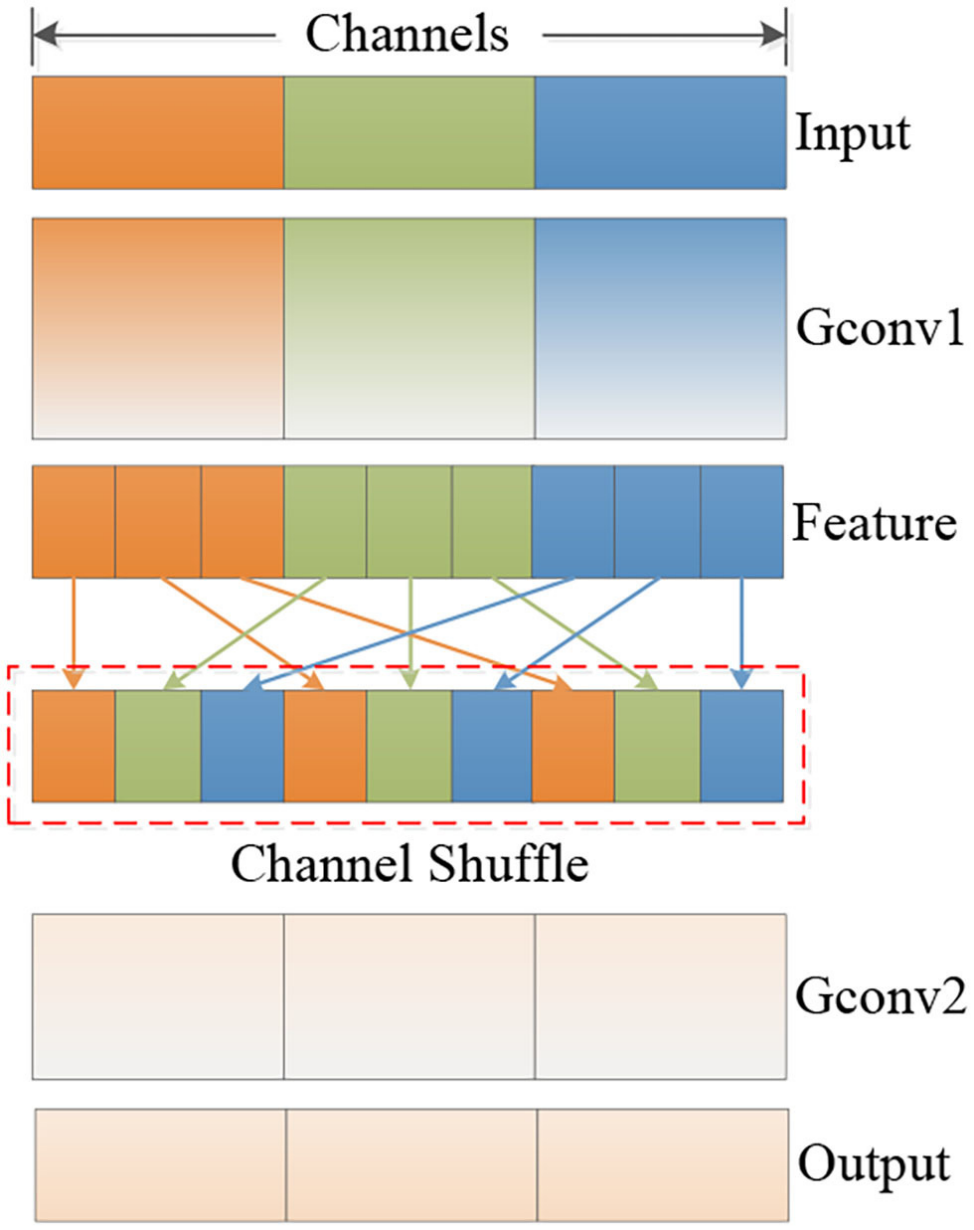
The structure of the ShuffleNet Unit.

With the development of computer vision technology, there is an increasing demand for running high-quality deep neural networks on mobile devices. Limited by the level of computing power, it is difficult for mobile devices to carry conventional convolution neural networks (CNNs) to deal with complete various tasks. In order to meet the requirements of applying deep neural networks on embedded and mobile terminals and maintaining superior performance, the MobileNet-v2 endows the model with remarkable feature extraction capabilities by stacking the Inverted Residual Block feature extraction structure. The specific method is to increase the dimensionality of input feature matrix through 1×1 convolution, and then use 3×3 deep separable convolution for feature learning, reduce the amount of model calculations, and, finally, decrease the dimensionality through 1×1 convolution and output after linear activation function. In order to give the model attention mechanism, MobileNet-v3 uses the Squeeze-and-Excitation module on the basis of MobileNet-v2 to optimize the feature learning ability of deep separable convolution. By assigning different weights to different channel features, the adaptability of the model to complex backgrounds is enhanced. Zhang et al. (2018) proposed ShuffleNet network to solve the problems of computing resources wasting and information interaction choked between group convolutions, and greatly reduce computational costs. The model mainly includes point-wise group convolution and channel shuffle operation, as is shown in Figure 2. Mobile terminal devices emphasize real-time, so it is necessary to speed up the inference process while maintaining the final accuracy. Commonly used inference acceleration methods, such as pruning existing models to reduce connection redundancy, quantification, and factorization, to reduce computational redundancy, and knowledge distillation of large models into small models are all accelerating and transforming existing models. ShuffleNet focuses on structural design to directly improve performance, and the core structure is more efficient.

In ShuffleNet-v1, floating-point operations per second (FLOPs) was used to measure the multiplication operation of convolution, and the design of this structure aims at reducing FLOPs. However, many factors that affect the model speed also include indicators, such as the memory access cost (MAC) and the level of parallelism. Other operations, such as data reading and writing, channel shuffling, etc., also consume certain time and affect the model inference speed. A large amount of point-wise group convolutions was used in ShuffleNet-v1, which increases MAC and reduces computational parallelism. In addition, a high degree of model fragmentation will significantly affect the speed of inference. The excessive use of element-wise operations, such as activation function, tensor addition, and offset addition, are not conducive to speed improvement. Based on the above criteria, Ma N. et al. (2018) redesigned the ShuffleNet Unit and proposed the idea of channel separation to replace the group convolution. The input feature is equally divided into two branches, and each branch maintains the same identity after separation. The 1×1 convolution is used to, instead, point-wise group convolution and maintained the same channel depth in a single branch. The reasoning speed is further improved by reducing element-wise operations, and channel shuffling is used to realize information interaction. Furthermore, the network structure can be scaled by controlling the number of convolution kernels, which could adjust the network width.

#### Structure of Proposed Model

In recent years, some progress has been made in the research of crop disease recognition based on attention mechanism (Karthik et al., 2020; Zeng and Li, 2020). The attention mechanism assigns high-contribution information to the larger weights while suppressing other irrelevant information through weights distribution, which is an effective method for model performance optimization. Different types of grape leaf diseases have relatively small differences, and the distinguishable fine-grained features are difficult to capture. Therefore, the effective attention mechanism to the characteristics of fine-grained lesions is the key to solving this problem.

**Figure 3:**
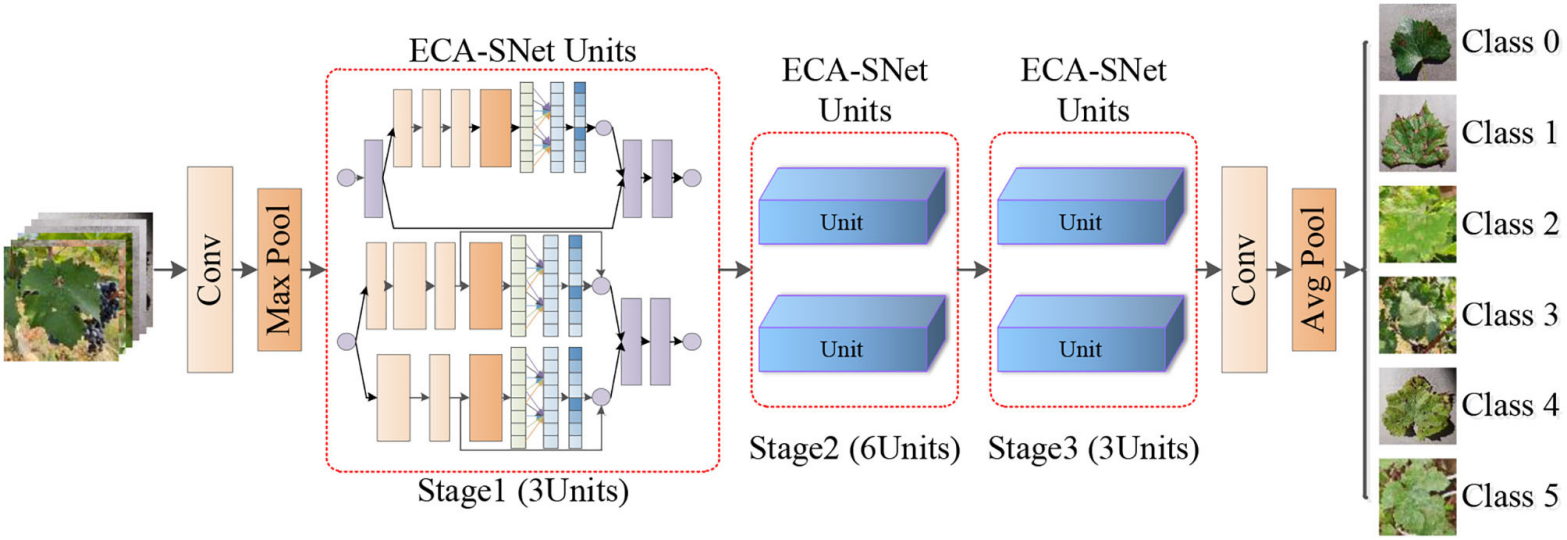
The overall structure of ECA-SNet.

Inspired by the above work, a fine-grained image recognition network for grape leaf diseases with a lightweight attention mechanism, namely ECA-SNet, is proposed in this paper. The main structure of the model includes three stages and a total of 12 ECA-SNet Units as is shown in Figure 3. First, the conventional convolution is implemented on the input image, and the Max pooling operation is used to reduce the size of output feature matrix to 1/4 of the input image, and then the characteristic information is learned through 12 ECA-SNet Units. Finally, the output features of the conventional convolutional layer and the pooling layer are sent to the fully connected layer for classification.

**Figure 4:**
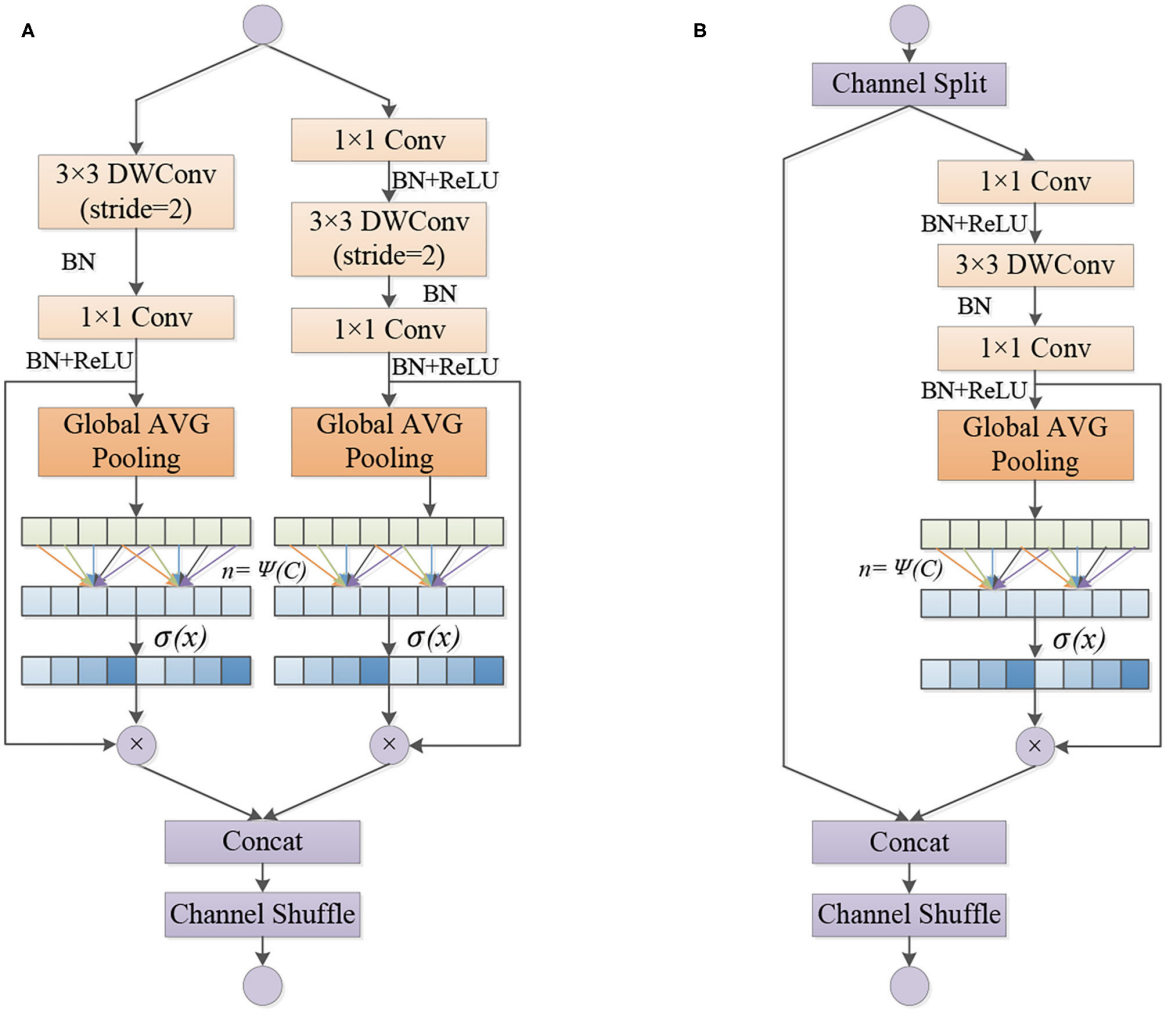
Detailed structure of the ECA-SNet Unit. **(A)** Unit 1. **(B)** Unit 2.

Wang et al. (2020) analyzed the conventional channel attention mechanism and found that the dimensionality reduction operation affects the performance of channel attention, and proper cross-channel interaction can significantly reduce the complexity of the model while maintaining efficient performance. Therefore, this paper adopts the strategy without dimensionality reduction in the design of basic structure of ECA-SNet Unit, as shown in Figure 4. There are two types of ECA-SNet Units. The module shown in Figure 4A (Unit 1) is the first unit of each Stage. The input feature matrix passing through two non-interacting branches and concatenating the two output feature matrices to doubled their depth. The ECA strategy is used in two branch structures, respectively. Figure 4B (Unit 2) module is the subsequent structure of each stage. First, the input feature matrix is equally divided into two groups. The main branch undergoes a series of operations and uses the ECA strategy, another branch output directly without operation and concatenating with the output of the main branch, and the depth of the feature matrix remain unchanged.

**Figure 5:**
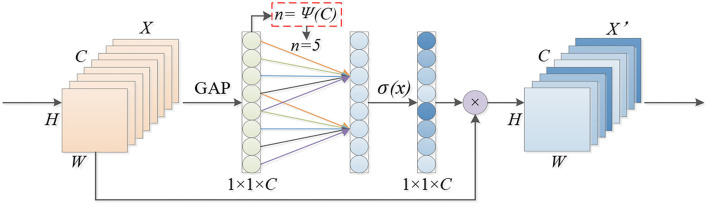
Efficient channel attention module.

The strategy without dimensionality reduction of cross-channel interaction that increases the revenue of the channel attention mechanism is shown in Figure 5. By considering each channel and its *n* neighborhoods, cross-channel interaction information is captured. The size of the convolution kernel *n* represents the coverage of cross-channel interaction, that is, the number of neighborhoods that participate in the attention prediction of a specific channel.

In order to avoid manual tuning, the method of adaptively selecting the one-dimensional convolution kernel size is used to determine the value of *n*. Conv1D is used to capture the cross-channel interaction; the size of *n* determines the coverage of the interaction. The number of *n* is related to the channel dimension *C*, and, in the case of fixed number group convolutions, the high-dimensional (low-dimensional) channel is proportional to the long-distance (short-distance) convolution. In the same way, the coverage of *n* of the cross-channel information interaction is also proportional to the channel dimension *C*, that is, the mapping relationship between *n* and *C* is shown in Equation (1):


(1) C=φ(n)

Based on the above analysis, it can be seen that *n* and *C* are in a non-linear proportion. As a kernel function, exponential family functions are widely used to deal with unknown mapping problems. So, the exponential function is used to approximate the mapping ϕ as shown in Equation (2). In addition, since the channel dimension is usually set to an integer power of 2, 2^(γ^*^n−b)^ is used instead of exp^(γ^*^n−b)^, and the mapping relationship of Equation (3) was obtained. In this paper, in order to reduce the time and computational costs of the training process and improve the friendliness of model training, the hyperparameters γ and *b* were set to 2 and 1, respectively. It can be seen that the function ϕ enables long-range interactions for large-sized channels.


(2) C=φ(n)≈exp(γ∗n−b)

(3) $$C=\varphi \left(n\right)=2^{\left(\gamma *n-b\right)}$$

Finally, given the channel dimension *C*, the size of convolution kernel *n* can be determined according to Equation (4), where |*m*|_*odd*_ represents the odd number closest to *m*.


(4) $$n=\Psi \left(C\right)={\left|\frac{\log_{2}\left(C\right)}{\gamma }+\frac{b}{\gamma }\right|}_{odd}$$

The detailed structure information of the model is shown in Table 2. Two different versions of networks, 0.5× and 1.0×, are designed according to the depth of the output feature matrix. Repeat represents the number of repetitions of a specific operation; multiple ECA-SNet Units are repeated in Stage1–Stage3. It should be noted that the first operation of each stage is Unit 1, which doubled the feature dimension, and is only used for the first layer in each stage, and Unit 2 is used for subsequent operations.

**Table 2:** Detailed structural information of ECA-SNet.

**Layer**	**Output size**	**Kernel size**	**Repeat**	**Output channel**	
				**0.5 × **	**1.0 × **
Input	224 × 224	–	–	3	3
Conv	112 × 112	3 × 3	1	24	24
MaxPool	56 × 56	3 × 3	1		
Stage1	28 × 28	–	3	48	116
Stage2	14 × 14	–	6	96	232
Stage3	7 × 7	–	3	192	464
Conv	7 × 7	1 × 1	1	1,024	1,024
avg pool	1 × 1	7 × 7	1	–	–
FC	–	–	1	6	6

## Experimental Results and Discussion

### Parameters Setting

In order to verify the performance of the ECA-SNet network, the Python language is used to build a model based on the Pytorch 1.7.1 deep learning framework, and the model is trained and tested on a GPU-equipped server. The detailed equipment configuration information of the test is shown in Table 3.

**Table 3:** Hardware and software environment.

**Configuration item**	**Value**
CPU	Intel^®^ Xeon(R) Gold 5217 CPU@3.00 GHz
GPU	NVIDIA Tesla V100 (32GB)
Operating system	Ubuntu 18.04.5 LTS 64
RAM	251.4GB
Hard disk	8TB

### Model Training Process

**Figure 6:**
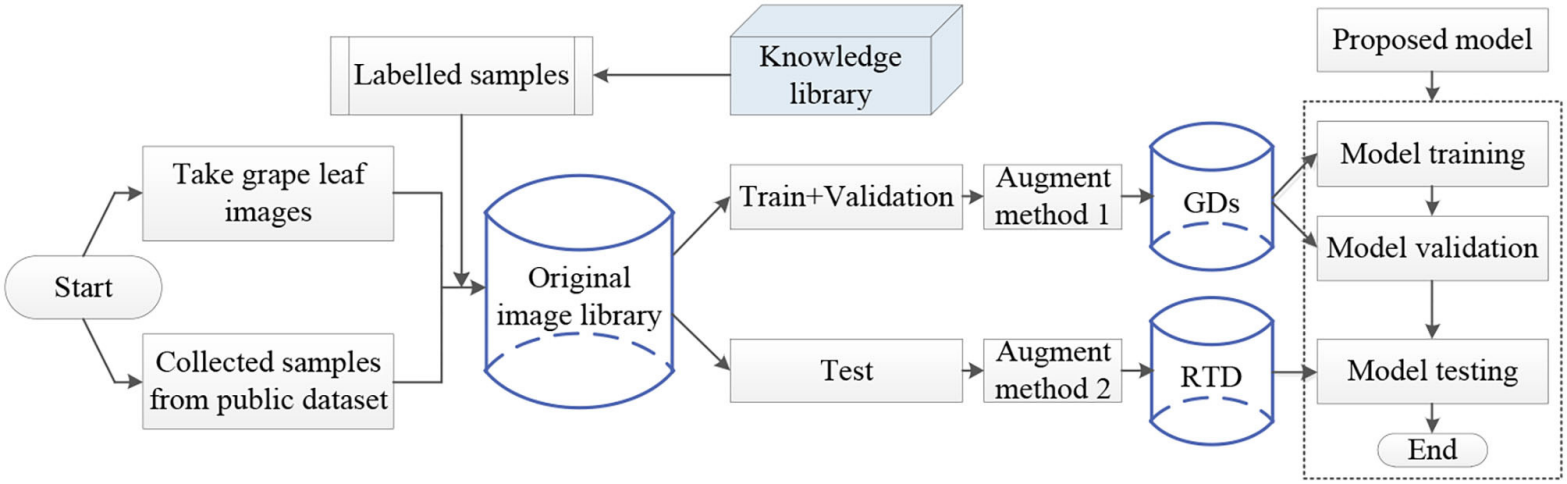
The overall flowchart of grape fine-grained disease image identification.

The experiment process of fine-grained image recognition of the grape disease is shown in Figure 6. First, images of grape leaves are collected from orchards and public data set, and disease categories are labeled based on expert experience and then, standardized the annotated disease images and divided the original image library into the training set, the validation set, the test set, and different methods are used to enhance the training set and the test set. Finally, the model is trained with FGDs and tested with RTD to identify the type of each disease.

### Weights Information Iterate Process

**Figure 7:**
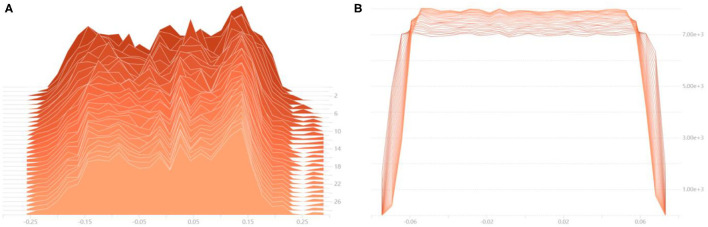
Histogram of weights numerical distribution of the convolutional layer. **(A)** The numerical distribution iteration process of the first convolutional layer, **(B)** The numerical distribution iteration process of the last convolutional layer.

In order to visualize the optimization process of the numerical distribution of the convolutional layer, the histogram of weight value distribution of part convolutional layers is drawn, which is shown in Figure 7. Figure 7A shows the iterative process of the weights information of the first convolutional layer of the network. The abscissa is the numerical change of the convolutional layer during the iteration process, and the ordinate is the number of iterations. It can be seen that the weights information with large contribution is gradually highlighted, indicating that the model has been continuously optimized during the training process. When iterating to the 25th epoch, the weights value basically no longer changes, indicating that the network training has tended to be saturated. Figure 7B is the tiled form of histogram of the last convolutional layer in the network. The abscissa represents the numerical information of the convolutional layer, and the ordinate represents the number of times the corresponding numerical value appears. With the training process proceeds, the data distribution tends to be concentrated. In order to keep the training process stable and convergent, the learning rate decays according to the cosine curve.

### Performance of Proposed Model

**Figure 8:**
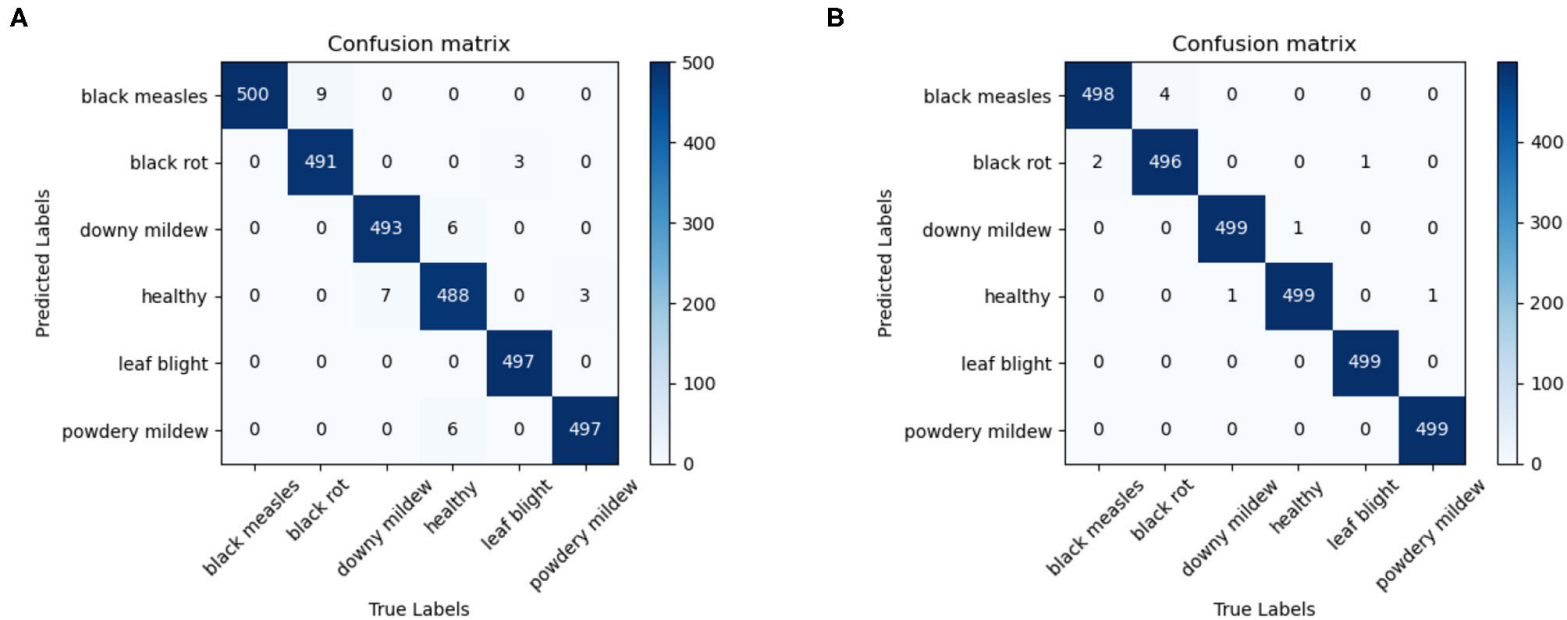
Confusion matrix of proposed ECA-SNet. **(A)** ECA-SNet_ 0.5 × **(B)** ECA-SNet_1.0 ×.

In order to evaluate the performance of the model, the proposed model is tested with RTD, and the confusion matrix of the 0.5× version and 1.0× version of ECA-SNet is shown in Figures 8A,B. Figures 8A,B show the classification performance of ECA-SNet, and the accuracy reached 98.86 and 99.66%, respectively. Among them, the main misclassification of 0.5× version of the model is misidentification of the Black rot as Black measles, and there is also a little misidentification between Downy mildew, Powdery mildew, and Healthy leaves. Compared with 0.5× version, ECA-SNet 1.0× has a higher recognition performance, and the error is further reduced. Accurate recognition of fine-grained images in a complex background poses a great challenge, but the false recognition of each category of the model in this paper is steadily maintained at a low level. The test results show that ECA-SNet can accurately perceive key areas and has strong robustness and stability for fine-grained image recognition of grape diseases in complex environments.

### Comparison of Proposed Model With Traditional Lightweight CNNs

In order to clarify the performance level of the model, the comparative test is conducted with multiple lightweight networks. Based on the confusion matrix, indicators, such as accuracy, precision, recall, and F1-score, are used to measure the comprehensive recognition performance of different grape diseases in each network. Accuracy, precision, recall, and F1-score are calculated from true positive (TP), false positive (FP), true negative (TN), and false negative (FN) results. The calculation of these indicators is shown in Equations (5–8):


(5) $$Accuracy=\frac{TP+TN}{TP+FP+TN+FN}$$

(6) $$Precision=\frac{TP}{TP+FP}$$

(7) $$Recall=\frac{TP}{TP+FN}$$

(8) $$F1-score=2\times \frac{Precision\times Recall}{Precision+Recall}$$

The performance indicators of ECA-SNet and other commonly used lightweight networks are compared, and the statistical results are shown in Table 4. MobileNet-v2 adopts the inverted residual structure, which effectively avoids the problem of model degradation. Deep separable convolution, the core of feature extraction, can greatly reduce the amount of parameters and calculations while ensuring accuracy. The RTD accuracy of MobileNet-v2 0.4 × can reach 95.23%, which proves that the bottleneck structure has a strong feature learning ability. It is worth noting that, although both the 0.4 × and 0.7 × versions of MobileNet-v2 can achieve acceptable recognition accuracy, they need more calculations compared with other lightweight networks, which has a certain impact on the running speed of the model. By introducing the channel attention mechanism and redesigning the time-consuming layer structure, MobileNet-v3 greatly reduces the amount of calculations and achieves an average F1-score slightly higher than MobileNet-v2. However, due to the adoption of the channel attention strategy that included the dimensionality reduction layer, it inevitably leads to the increases of the parameters. The ShuffleNet-v2 network optimized the ShuffleNet structure based on criteria, such as optimal MAC, reduced network fragmentation, and reduced element-wise operations. Since the network failed to pay attention on the fine-grained information of the key areas of grape leaves when extracting features, the ShuffleNet-v2 test results performed poorly. Additionally, it can be seen from Table 4 that the higher complexity of the relevant network has a certain improvement in the effect of disease identification. It is because the increase of feature extraction layer structure enables the network to learn more relevant features, but this also leads to a rapid increase of model volume.

**Table 4:** Performance comparison of the proposed ECA-SNet against the classical lightweight CNNs.

**Model**	**Accuracy/%**	**Average Precision/%**	**Average Recall/%**	**Average F1-score**	**FLOPs/M**	**Params/M**
MobileNet-v3 small_0.75 ×	94.76	95.28	94.76	0.950	40.7	0.89
ShuffleNet-v2_0.5 ×	92.46	92.81	92.46	0.926	41.5	0.35
MobileNet-v2_0.4 ×	95.23	92.25	95.23	0.937	81.5	0.39
**ECA-SNet_0.5 × **	**98.86**	**98.86**	**98.86**	**0.988**	**37.4**	**0.31**
MobileNet-v3 large_0.75 ×	96.70	96.83	96.70	0.967	146.1	2.42
ShuffleNet-v2_1.0 ×	95.20	95.30	95.20	0.952	147.8	1.26
MobileNet-v2_0.7 ×	95.93	96.03	95.93	0.959	178.6	1.12
**ECA-SNet_1.0 × **	**99.66**	**99.66**	**99.66**	**0.996**	**125.6**	**1.08**

*Bold values indicate best results under each index*.

ECA-SNet generates high-efficiency channel attention by adaptively selecting the size of a one-dimensional convolution kernel on the basis of ShuffleNet-v2 and avoiding dimensionality reduction operations. The channel interaction strategy greatly improved the performance of channel attention, making ECA-SNet have the accurate recognition performance. The test accuracy of ECA-SNet 0.5 × and ECA-SNet 1.0 × with the RTD reaches 98.86 and 99.66%, respectively, which are higher than other networks of the same magnitude and have the least amount of parameters and computational costs. The test results show that avoiding dimensionality reduction and proper cross-channel interaction is very important for learning efficient channel attention.

### Network Attention and Fine-Grained Visualization

The evaluation of model performance through common indicators lacks intuitive display, and it is difficult to understand which part of the input image the model relies on to make decisions. In order to understand and analyze the network structure and visually display the model decision-making basis, the attention heat map visualization method is used to display the attention area. Table 5 shows the attention heat map of ECA-SNet and other commonly used lightweight models. Sample images of healthy, downy mildew, black rot, powdery mildew, and leaf blight are randomly selected for testing. The red mark in the original image in Table 5 is the annotation information of the diseased area on the grape leaves by the expert. According to the visualization results, it can be seen that the location of the key fine-grained features of grape disease images in a complex background is difficult to determine, and neither ShuffleNet nor MobileNet can accurately focus on the key feature regions. The MobileNet-v2 network pays attention to much background information, which leads to insufficient feature learning. On account of MobileNet-v3 introduction of a channel attention mechanism, it plays a certain role in the key area of feature learning and reduces the attention of background information, but the effect is still poor. The ECA-SNet proposed in this paper can distinguish the foreground and the background and accurately locate the key areas. The leaf diseased area is strongly activated as the decision-making basis. In the recognition of diseased leaves with a purity background, each network can focus on the diseased area to varying degrees and has remarkable recognition ability. Compared with other networks, ECA-SNet can more comprehensively focus on lesion areas in different locations and has more superior decision-making capability.

**Table 5:** Visualization of attention heat map of grape leaf diseases.

**Class**	**Original image**	**ShuffleNet-v2 0.5 × **	**MobileNet-v2 small 0.4 × **	**MobileNet-v3 small 0.75 × **	**ECA-SNet 0.5 × **
Healthy	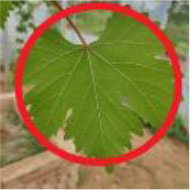	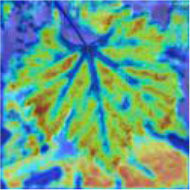	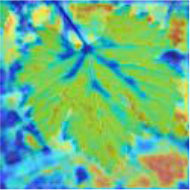	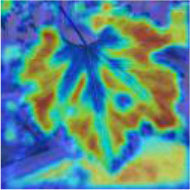	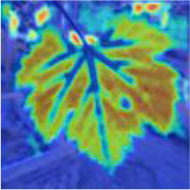
Downy mildew	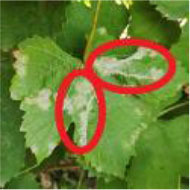	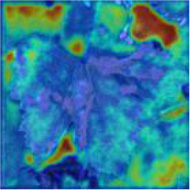	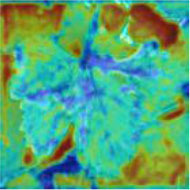	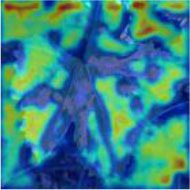	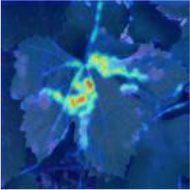
Black rot	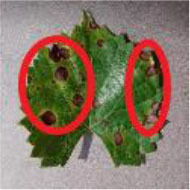	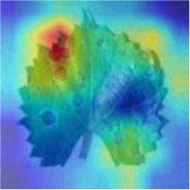	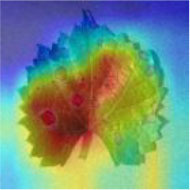	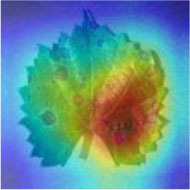	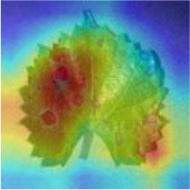
Powdery mildew	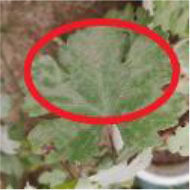	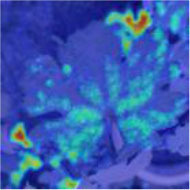	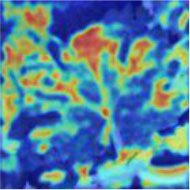	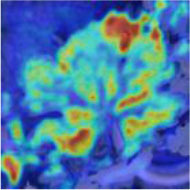	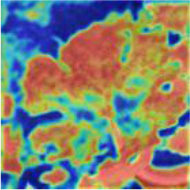
Leaf blight	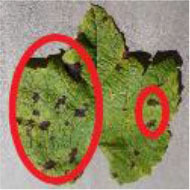	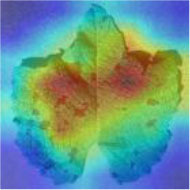	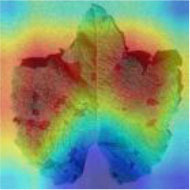	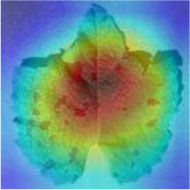	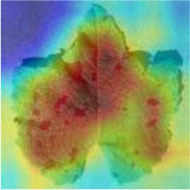

The diseased spots of grape leaves are usually scattered randomly in different positions on the leaves; the shape, size, and density of the diseased spots will affect Grad-CAM++ (Chattopadhyay et al., 2017). Therefore, in order to further display the fine-grained information of the network focus area, the guided back propagation and Grad-CAM++ were dot multiplication to obtain visual feature maps. The feature maps obtained above have both high resolution and category separability. Some example image test results are shown in Table 6. The red mark in the original image in Table 6 is the annotation information of the region of interest (diseased area) on the grape leaves by the expert. The feature maps of ShuffleNet-v2 are sensitive to large areas of lesions and can accurately locate the area of lesions. When the lesions become smaller and their locations gradually disperse, the fine-grained information extraction capability of the model is greatly declined. The fine-grained information extracted by MobileNet-v2 has much redundancy, which affects the accurate judgment of the model. MobileNet-v3 reduces redundancy, but the richness of fine-grained information is also reduced and fails to accurately locate the key information for classification decisions. According to Table 6, it can be found that ECA-SNet has excellent adaptability to lesion features; the key fine-grained information is comprehensive, and the information redundancy is low. The model in this paper can accurately locate different lesion shapes, positions, and densities and can make accurate classification decisions accordingly. The above test results show that the model in this paper fully considers the characteristics of disease spots and model structure, and the performance of grape leaf disease recognition is improved significantly.

**Table 6:** Fine-Grained feature visualization of grape leaf diseases.

**Class**	**Original image**	**ShuffleNet-v2 0.5 × **	**MobileNet-v2 small 0.4 × **	**MobileNet-v3 small 0.75 × **	**ECA-SNet heatmap**	**ECA-SNet 0.5 × **
Black measles	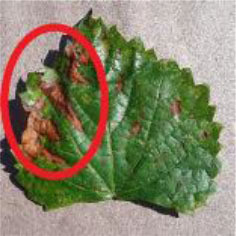	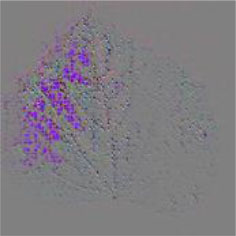	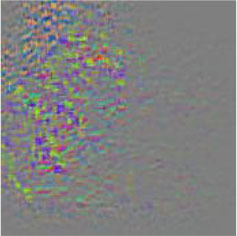	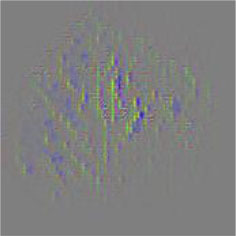	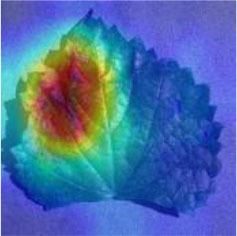	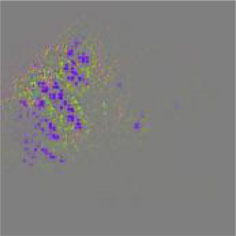
Black rot	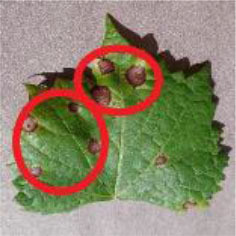	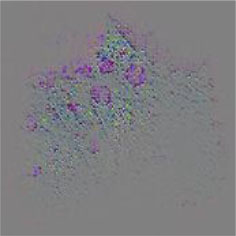	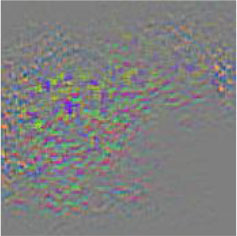	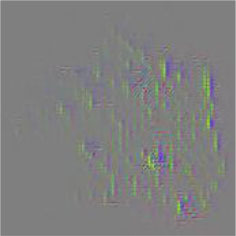	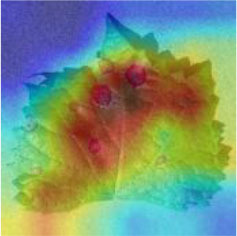	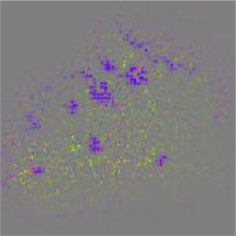
Leaf blight	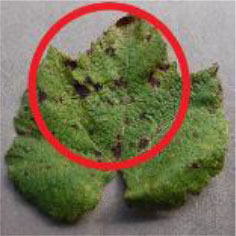	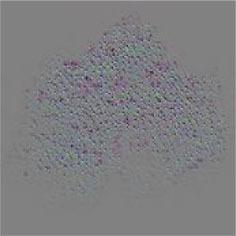	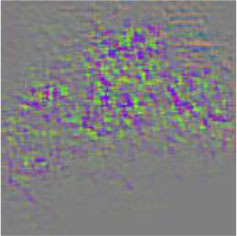	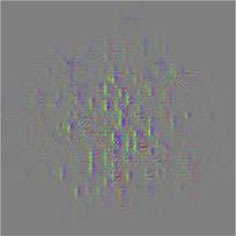	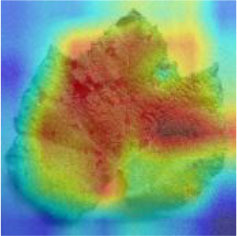	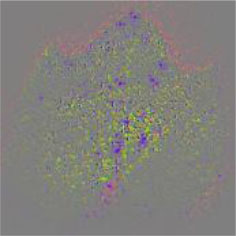

## Conclusion

A fine-grained image recognition model for grape diseases with an improved lightweight channel attention mechanism is proposed in this paper, which provides technical support for dynamic and efficient management of orchard grape diseases. Based on mobile devices, 3,388 images of grape leaf diseases are collected in the field, and 3,479 images are obtained from public data sets. By using image-enhancement techniques, the FGDs containing 62,670 images are generated. First, on the basis of ShuffleNet, a cross-channel interaction strategy without dimensionality reduction is used to make the model have efficient channel attention. Second, the layer structure is reduced in different stages to build an efficient ECA-SNet with a less parameter. Ultimately, the cross-channel coverage is determined by adaptively selecting the one-dimensional convolution kernel, which reduces the calculation costs while maintaining efficient channel attention performance. The proposed model is trained with FGDs and has been tested with RTD. The comparative experiments, including various performance evaluation indicators and process visualization, are carried out.

Through the experimental results, it can be seen that the model proposed in this paper achieves the best recognition effect under the condition of extremely low calculation and parameters, with an accuracy of 98.86% and the F1-score of 0.988. Means, such as visualization, also show the superior performance of the model and realize the efficient performance of fine-grained disease images identification of grape leaves. The above work laid the theoretical foundation for the next development of automatic inspection equipment for disease identification and real-time orchard grape disease information acquisition.

## Data Availability Statement

The raw data supporting the conclusions of this article will be made available by the authors, without undue reservation.

## Author Contributions

PW, YM, and TN collected data. PW designed and performed the experiment, analyzed data, trained algorithms, and wrote the manuscript. BL, SY, DH, and QG conceived the study and participated in its design. All the authors contributed to the article and approved the submitted version.

## Funding

This work was funded by the Key Research and Development Program of Shaanxi under Grants Nos. 2021NY-138 and 2019ZDLNY07-06-01.

## Conflict of Interest

The authors declare that the research was conducted in the absence of any commercial or financial relationships that could be construed as a potential conflict of interest.

## Publisher's Note

All claims expressed in this article are solely those of the authors and do not necessarily represent those of their affiliated organizations, or those of the publisher, the editors and the reviewers. Any product that may be evaluated in this article, or claim that may be made by its manufacturer, is not guaranteed or endorsed by the publisher.
